# Physician Use of Multiple Criteria to Diagnose Periprosthetic Joint Infection May Be Less Accurate Than the Use of an Individual Test

**DOI:** 10.7759/cureus.31418

**Published:** 2022-11-12

**Authors:** Carl Deirmengian, Alex McLaren, Carlos Higuera, Brett R Levine

**Affiliations:** 1 Orthopaedic Surgery, The Rothman Orthopaedic Institute, Philadelphia, USA; 2 Orthopaedic Surgery, Thomas Jefferson University, Philadelphia, USA; 3 Orthopaedic Surgery, University of Arizona College of Medicine - Phoenix, Phoenix, USA; 4 Orthopaedic Surgery, Cleveland Clinic Florida, Weston, USA; 5 Orthopaedic Surgery, Rush University Medical Center, Chicago, USA

**Keywords:** diagnosis, clinical practice guidelines, periprosthetic joint infection, total hip arthroplasty: tha, knee arthroplasty

## Abstract

Introduction

Multiple-criterion scoring systems for periprosthetic joint infection (PJI) can be algorithmically implemented in research, diagnostically outperforming individual tests. This improved performance may be lost in the practice setting, where clinicians rarely utilize strict algorithms. The ability of physicians to interpret multiple criteria for PJI and confront the complexity of combining them into a final diagnosis has never been studied. This study assessed the diagnostic characteristics of physicians using multiple criteria to diagnose PJI and compared the physicians’ diagnostic accuracy to that of individual tests.

Methods

A total of 12 physicians, including academic arthroplasty surgeons (N=4), community arthroplasty surgeons (N=4), and infectious disease (ID) specialists (N=4) were asked to use their routine clinical diagnostic practice to assign a diagnosis to 277 clinical vignettes using multiple preoperative laboratory criteria for PJI. The undecided rate, interobserver agreement, and accuracy of physicians were characterized relative to the 2013 Musculoskeletal Infection Society (MSIS) gold standard and compared to the accuracy of each individual laboratory test for PJI.

Results

Physicians interpreting multiple criteria for PJI demonstrated high undecided diagnosis rates (mean=23.5%), poor interobserver agreement (kappa range=0.49-0.63), and mean accuracy of 90.8% (range:85.8%-97.4%) compared to the 2013 MSIS gold standard. The group of academic arthroplasty surgeons had a lower rate of undecided diagnoses than community arthroplasty surgeons (16.2% vs. 29.1%; p<0.0001) or ID specialists (16.2% vs. 25.1%; p<0.0001). Academic arthroplasty surgeons also exhibited a higher interobserver agreement than community arthroplasty surgeons (kappa = 0.63 (95%CI:0.59-0.68) vs. 0.49 (95%CI:0.44-0.54)).

Mean physician accuracy (90.8%) was inferior to the alpha-defensin laboratory test (96.0%;p=0.0034) and the alpha-defensin lateral-flow test (94.6%;p=0.036), comparable to synovial fluid white blood cells (SF-WBC) (93.3%;p=0.17) and synovial fluid polymorphonuclear cell % (SF-PMN%) (94.0%;p=0.11), and superior to the erythrocyte sedimentation rate (ESR) (86.2%;p<0.0001) and C-reactive protein (CRP) (84.6%;p<0.0001). Only two academic arthroplasty surgeons in this study were able to outperform every individual test for PJI by combining multiple criteria to make a diagnosis.

Conclusion

Although multiple-criterion scoring systems may outperform individual tests for diagnosing PJI in the research setting, it appears that the complexity of using multiple tests to diagnose PJI causes indecision and variability among physicians. Physician use of multiple preoperative criteria to diagnose PJI is less accurate than the strict algorithmic calculation of the diagnosis as achieved in research. In fact, most physicians in this study would have improved their diagnostic accuracy for PJI by simply utilizing a single good test to make the diagnosis, instead of trying to combine multiple tests into a decision. We propose that less complex diagnostic criteria should be explored for routine clinical utilization.

## Introduction

Standards to diagnose periprosthetic joint infection (PJI) have made a fundamental shift away from complete reliance on culture toward strategies that include the potential to diagnose culture-negative PJI using multiple criteria [1-3]. Several authoritative bodies, including the Musculoskeletal Infection Society (MSIS) [2], the International Consensus Meeting (ICM) [3], the European Bone and Joint Infection Society (EBJIS) [1], and the Infectious Diseases Society of America (IDSA) [4], have released their own versions of a PJI scoring system over the past decade. The intent of these scoring systems is two-fold: 1) to provide investigators with a common tool for standardization in research and 2) to provide a diagnostic clinical practice guideline (CPG) for routine patient care.

Although PJI scoring systems have been an absolute triumph for infection research, it is not clear that these scoring systems can be easily translated to routine patient care, as they exhibit several of the well-described barriers to CPG adoption and correct usage [5-9]. First, there remains significant expert disagreement [5,6] regarding multicriteria definitions, with as low as 68% consensus on the definition of PJI at the 2018 ICM [3]. Second, the definitions of PJI have substantial complexity [5,6], with multiple categories, multiple specific criteria, and their subsequent combination to create a score [1-3]. Third, several societies have presented conflicting versions of these definitions, which have changed frequently even over the last decade [1-4]. Fourth, laboratory-reported normal ranges are frequently not compatible with PJI-optimized testing thresholds [10], requiring unit conversions and alternative PJI-optimized interpretation.

While the complexities in utilizing a PJI scoring system can be managed effectively in research using spreadsheets and rules-based algorithms, clinicians attempting to combine multiple criteria for PJI in routine practice may find them overwhelming and confusing [11]. Supporting this concern, the existing literature on clinical practice reveals poor adoption of diagnostic PJI guidelines [11-13] and evidence of poor interobserver diagnostic agreement [14] between expert clinicians using a PJI scoring system.

The ability of physicians to interpret multiple criteria for PJI and confront the complexity of combining them into a final diagnosis has never been studied. The purpose of this study was to assess the diagnostic characteristics of physicians using multiple criteria to diagnose PJI and compare the physicians’ diagnostic accuracy to that of individual tests.

## Materials and methods

This survey study presented clinical vignettes, including laboratory and clinical data, to physicians who were asked to diagnose PJI. The internal review board (IRB) assessed the study as exempt.

Surveyed physicians

A total of 12 physicians who routinely care for arthroplasty patients participated in this study, including 4 fellowship-trained academic arthroplasty surgeons (academic surgeons), 4 community arthroplasty surgeons (community surgeons), and 4 infectious disease specialists (ID specialists). Within each physician group, all four physicians were from different states and different institutions to minimize institutional or geographic bias.

All 12 physicians were asked to review the clinical vignettes and combine the multiple laboratory and clinical data provided to diagnose PJI as they usually would in routine clinical care. The physicians were not alerted to the purpose of the study and were not specifically provided with any multicriteria tool for diagnostic use, as one purpose of this study was to assess their existing capability to combine multiple diagnostic and clinical data into a final diagnosis. Furthermore, the physicians were not provided with any scoring system’s specific diagnostic thresholds for individual test positivity. The survey period, conducted in an online setting, was conducted from May 2020 through January 2021.

Clinical vignettes

In a previous multi-institutional study audited by a contracted research organization, the diagnostic performance of a biomarker for chronic PJI was evaluated for Food and Drug Administration (FDA) review [15]. We utilized the data from that previous study in the current survey study due to the following favorable cohort characteristics: 1) The availability of multiple laboratory test results for each case that reflects practicing arthroplasty surgeons’ actual diagnostic work-up, 2) Data acquisition through a contracted research organization that periodically audited results to assure data integrity, 3) Inclusion of patients from three arthroplasty centers over a period of 18 months to reduce institutional biases, 4) Assignment of a gold-standard 2013 MSIS criteria definition of PJI to each case by a panel of three surgeon judges who agreed to the presence of sufficient data and the final diagnosis, and 5) Exclusion of cases with insufficient criteria to assign a diagnosis based on the 2013 MSIS definition.

This previous study included a prospective sub-cohort of 288 patients with good-quality synovial fluid samples and associated clinical information and laboratory data [15]. Of these 288 cases, 11 cases were excluded in this study due to the presence of a sinus tract and thus a clinically obvious diagnosis, and 3 cases were excluded due to the availability of only one preoperative minor criterion, leaving 277 cases for this survey study. These 277 patient cases were organized into clinical vignettes that were utilized as cases for the current study. Of 277 vignettes in this study, 42 were given the diagnosis of PJI, and 235 were aseptic as determined by the three-surgeon judging panel using the 2013 MSIS definition of PJI in the previous study (Table 1) [15]. The laboratory and clinical data for each case were compiled into a paragraph to create 277 clinical vignettes. Although the gold-standard diagnosis previously assigned to each case was based on both preoperative and postoperative data, including tissue culture and histology, the clinical vignettes used in this survey only presented preoperative data to simulate real, clinical preoperative decision-making.

**Table 1:** Laboratory values associated with patient cohorts Characteristics of the cohort from the previous biomarker study by Deirmengian et al. [15], which was utilized in this study MSIS=Musculoskeletal Infection Society; ESR=Erythrocyte Sedimentation Rate; CRP=C-reactive protein; SF-WBC=Synovial fluid white blood cells; SF-PMN%-synovial fluid polymorphonuclear cell %

MSIS Diagnosis	N	ESR (mm/hr) Median	CRP (mg/L) Median	SF-WBC (cells/ul) Median	SF-PMN% (%) Median
Aseptic	235	10	4	611	14
PJI	42	56	38	27,940	93

Included in the laboratory data presented in each clinical vignette were all traditional preoperative laboratory values actually ordered as part of the patient’s standard of care work-up, possibly including erythrocyte sedimentation rate (ESR), C-reactive protein (CRP), synovial fluid white blood cells (SF-WBCs), synovial fluid polymorphonuclear cell percentage (SF-PMN%), and the synovial fluid culture result with species. Among 277 cases, 75.1% included all five preoperative test results, 91.0% included at least four preoperative test results, and 98.9% included three or more preoperative test results. Three cases had only two preoperative laboratory tests available, as the patients had negative serology (negative ESR and CRP) and were not aspirated preoperatively. Included in the clinical data presented with each clinical vignette were patient age, gender, arthroplasty (knee/hip), laterality, concurrent antibiotic treatment, and co-morbidities classified as systemic inflammatory disease or immunocompromise. Results of the alpha-defensin test were not included in the clinical vignettes, as alpha-defensin was not included in the 2013 MSIS definition of PJI [2].

Survey methods

All 277 clinical vignettes were presented on the SurveyMonkey platform (Surveymonkey.com) and divided into three individual surveys to minimize survey fatigue. In stage I of the survey study, physicians were asked to choose a diagnosis of PJI, Aseptic, or Undecided, based on the presented vignette, to measure the rate of initially undecided diagnoses and assess interobserver agreement. In stage II of the survey study, performed greater than two weeks after stage I, physicians were asked to choose a diagnosis of PJI or Aseptic for cases they previously diagnosed as undecided, resulting in all vignettes having a clinical diagnosis.

Diagnostic performance of individual laboratory tests

The previous study from which the clinical vignettes were generated included several individual laboratory criteria for PJI, including serology (ESR, CRP), traditional synovial fluid testing (SF-WBC and SF-PMN%), and modern biomarker testing (alpha-defensin laboratory test and lateral flow test). Cutoffs for traditional tests were based on the 2013 MSIS criteria for PJI [2]. The alpha-defensin laboratory test was interpreted as reported by the laboratory. The alpha-defensin lateral flow test (Zimmer Biomet, Warsaw, IN) was interpreted by each institution involved in the multicenter trial, following the manufacturer's direction to read the lines generated on the lateral-flow device.

Data analysis and statistics

Randolph’s free marginal kappa proportion of agreement was calculated to assess the interobserver agreement within groups in this study, including three categories (infected, aseptic, undecided) and four raters (per physician group). Given the importance of the diagnosis of PJI to the patient and medical decision-making, a minimum acceptable kappa coefficient of 0.9 was used as a diagnostic standard [16], below which agreement was considered poor. After physicians chose a final diagnosis for vignettes previously given an undecided diagnosis, all results were analyzed to determine the diagnostic performance of physicians and physician groups.

The comparison of physician diagnostic performance versus individual laboratory test performance was assessed using two-by-two contingency tables and the two-sided Chi-squared test to compare proportions (GraphPad Software, San Diego, CA). Correction for multiplicity was not performed, as the laboratory tests being compared were preplanned given their inclusion as criteria for PJI.

## Results

Diagnostic characterization of physicians interpreting multiple criteria for PJI

The rate of undecided diagnoses among 12 physicians interpreting multiple diagnostic criteria to diagnose PJI in this study (Table 2) was 23.5% (range: 5.4 to 44.0%), with academic surgeons as a group choosing an undecided diagnosis less frequently than community surgeons or ID specialists (16.2% vs. 29.1%, and 16.2% vs. 25.1%; both p<0.0001). Among 140 cases with a negative result for all five laboratory tests (ESR, CRP, SF-WBC, SF-PMN%, and culture), the mean undecided rate across all physicians was 16.3%.

**Table 2:** Undecided diagnoses by physician and physician group The rate of undecided diagnoses among physicians and physician groups using multiple criteria to diagnose periprosthetic joint infection ID=infectious disease

Group		Undecided (N)	Total Diagnoses	Undecided Rate	Significance
Academic Surgeons		180	1108	16.2%	Comparator
	Physician 1	45	277	16.2%	
	Physician 2	15	277	5.4%	
	Physician 3	17	277	6.1%	
	Physician 4	103	277	37.2%	
Community Surgeons		322	1108	29.1%	<0.0001
	Physician 1	94	277	33.9%	
	Physician 2	60	277	21.7%	
	Physician 3	62	277	22.4%	
	Physician 4	106	277	38.3%	
ID Physicians		278	1108	25.1%	<0.0001
	Physician 1	122	277	44.0%	
	Physician 2	54	277	19.5%	
	Physician 3	28	277	10.1%	
	Physician 4	74	277	26.7%	

The interobserver agreement in diagnosing PJI using multiple diagnostic criteria (Table 3) revealed generally a poor agreement between physicians when interpreted in terms of diagnostic testing, with free-marginal kappa values of 0.63 (95%CI:0.59-0.68), 0.49 (95%CI:0.44-0.54), and 0.61 (95%CI: 0.56-0.66) for academic surgeons, community surgeons, and ID specialists, respectively.

**Table 3:** Inter-physician agreement by physician group The free-marginal kappa coefficient for inter-physician agreement Kappa<0.9 is considered poor agreement for a clinically important test.

Group	Marginal Free Kappa	95% CI
Academic Surgeons	0.63	0.59-0.68
Community Surgeons	0.49	0.44-0.54
ID Specialists	0.61	0.56-0.66

The mean sensitivity, specificity, and accuracy of physicians interpreting multiple criteria (Table 4) to diagnose PJI was 98.4%, 89.5%, and 90.9%, relative to the 2013 MSIS gold standard. The total number of correct diagnoses among academic surgeons (94.1%; 1043/1108) was greater than that of community surgeons (90.3%; 1000/1108; p = 0.0007) and ID physicians (88.2%; 977/1108; p < 0.0001), mostly attributable to greater specificity. Of 201 aseptic cases with zero positive MSIS criteria, the total false-positive diagnosis rate among academic surgeons (2.4%) was lower than that of both community surgeons (6.2%; p<0.0001) and ID specialists (7.7%; p<0.0001).

**Table 4:** The diagnostic performance of physicians and physician groups diagnosing periprosthetic joint infection using multiple criteria The diagnostic characteristics of physicians and physician groups in this study Accuracy=the total number of correct diagnoses as a percentage of all cases

Group		Sensitivity	Specificity	Accuracy	Significance of Accuracy
Academic Surgeons		98.2%	93.4%	94.1%	Comparator
	Physician 1	100.0%	97.0%	97.5%	
	Physician 2	100.0%	94.9%	95.7%	
	Physician 3	95.2%	97.9%	97.5%	
	Physician 4	97.6%	83.8%	85.9%	
Community Surgeons		98.8%	88.7%	90.3%	p=0.0007
	Physician 1	95.2%	93.6%	93.9%	
	Physician 2	100.0%	83.4%	85.9%	
	Physician 3	100.0%	92.3%	93.5%	
	Physician 4	100.0%	85.5%	87.7%	
ID Physicians		98.2%	86.4%	88.2%	p<0.0001
	Physician 1	97.6%	88.1%	89.5%	
	Physician 2	97.6%	86.0%	87.7%	
	Physician 3	97.6%	85.5%	87.4%	
	Physician 4	100.0%	86.0%	88.1%	

Physician accuracy versus individual laboratory test accuracy relative to the 2013 MSIS criteria for PJI

Mean physician diagnostic accuracy (90.8%) was inferior to the alpha-defensin laboratory test (96.0%; p=0.0034) and the alpha-defensin lateral-flow test (94.6%;p=0.036), equivalent to SF-WBC (93.3%;p=0.17) and SF-PMN% (94.0%;p=0.11), and superior to the ESR (86.2%;p<0.0001) and the serum CRP (84.6%;p<0.0001). The alpha-defensin tests, SF-WBC and SF-PMN%, ranked as being more accurate than the majority of physicians using multiple preoperative criteria to diagnose PJI (Table 5).

**Table 5:** Accuracy rank order of physicians and individual laboratory tests for the diagnosis of periprosthetic joint infection. Diagnostic accuracy rank order of all physicians and individual laboratory tests in this study. Laboratory tests are in bold text. AS=Academic surgeon; CS=Community surgeon; ID=Infectious disease specialist; MSIS=Musculoskeletal Infection Society; AD=Alpha-defensin; ESR=Erythrocyte sedimentation rate; CRP=C-reactive protein; SF-WBC=Synovial fluid white blood cells; SF-PMN%=synovial fluid polymorphonuclear cell %

Physician or test	Accuracy	Rank
AS1	97.5%	1
AS3	97.5%	1
AD Laboratory	96.0%	3
AS2	95.7%	4
AD Lateral Flow	94.6%	5
SF-PMN%	94.0%	6
CS1	93.9%	7
CS3	93.5%	8
SF-WBC	93.3%	9
ID1	89.5%	10
ID4	88.1%	11
CS4	87.7%	12
ID2	87.7%	12
ID3	87.4%	14
ESR	86.2%	15
AS4	85.9%	16
CS2	85.9%	16
CRP	84.6%	18

## Discussion

There has been minimal research assessing the adoption and proper use of PJI scoring systems among physicians caring for arthroplasty patients [11-13]. This study assessed physicians’ routine practice of interpreting multiple criteria to diagnose PJI, demonstrating high undecided diagnosis rates, poor interobserver agreement, and diagnostic decision-making that often did not match the formally calculated diagnosis using the 2013 MSIS scoring system. We did not direct any of the physician experts to use a diagnostic scoring system to make their diagnosis, however, all physician experts were fully knowledgeable about PJI scoring systems. Their diagnostic results imply that the physicians either did not use a scoring system or did not use one correctly. These results raise concern that PJI scoring systems, which are dependent on multiple criteria, have not translated effectively to the clinical care setting. In fact, the majority of physicians participating in this study would have had equivalent or superior accuracy by trusting one good laboratory test instead of trying to interpret multiple criteria for PJI. We propose that less complex diagnostic criteria should be explored for routine clinical utilization.

This study demonstrated that physicians interpreting multiple criteria for PJI have high rates of indecision (range: 5.4% to 44.0%) and poor interobserver agreement (kappa range: 0.49 to 0.63). Additionally, this study demonstrated lower undecided rates (mean=16.2%) and higher interobserver agreement (0.63) among academic arthroplasty surgeons. These findings confirm the results of a smaller but similar study by Amanatullah et al., which assessed the diagnostic characteristics of physicians forming a diagnosis based on the 2013 MSIS criteria [14]. The authors reported that the principal investigator exhibited a 19% (22/117) undecided diagnosis rate using multiple preoperative criteria to diagnose PJI. They also demonstrated an overall physician interobserver agreement coefficient of 0.62, with arthroplasty surgeons (kappa=0.80; 95%CI:0.75 to 0.84) showing greater interobserver agreement than ID specialists (kappa=0.55; 95%CI: 0.51 to 0.60). These findings, in combination with those of the current study, suggest that using multiple criteria without the algorithmic direction of a research spreadsheet is not straightforward. Furthermore, the apparent diagnostic advantage among academic arthroplasty surgeons as a group, in both studies, is explained by their likely increased familiarity with the PJI scoring system, leading to lower indecision, greater interobserver agreement, and a higher likelihood to match a PJI scoring system.

The current study is the first to assess the performance of physicians diagnosing PJI with multiple criteria as they would in routine practice, with or without the algorithmic guidance of a formal scoring system. The mean physician diagnostic accuracy relative to the 2013 MSIS criteria was 90.8%, with academic surgeons exhibiting higher accuracy (94.1%) than both community surgeons (90.1%) and ID specialists (88.2%) [2]. These results demonstrate that physicians diagnosing PJI with multiple criteria often arrive at a diagnosis that does not match the algorithmically calculated PJI scoring system diagnosis. The trend favoring academic arthroplasty surgeons is again likely due to their increased familiarity with formally recommended testing thresholds and the manner in which to combine tests to match a PJI scoring system. The gap between physician diagnoses and the diagnosis of a PJI scoring system is not at all surprising considering the complexity of scoring systems and their well-described barriers to adoption [5,6], including low expert consensus [3], competing versions of the scoring system [1-3], multiple rules [1-4], and ambiguity in the laboratory test thresholds [10]. Therefore, while PJI scoring systems provide an objective standard by which to diagnose PJI in research, they may be too complex for routine use in clinical medicine [5,6].

Finally, this study has demonstrated that several preoperative laboratory tests can match the 2013 MSIS criteria with equivalent or improved accuracy compared to physicians trying to interpret multiple tests. While the goal of combining multiple tests to surpass the accuracy of any individual test has been achieved with PJI scoring systems in research, it appears that physicians trying to combine multiple tests may not fare as well. The alpha-defensin laboratory and lateral flow tests both demonstrated higher accuracy in predicting the 2013 MSIS diagnosis than physicians in this study, which is especially notable considering it is the only test in this study that does not influence the 2013 MSIS diagnosis of PJI. The main advantage of the alpha-defensin test is that its accuracy is transferred with ease to clinical physicians, given that it is standardized and provides a normal versus abnormal result in terms of PJI, which frees the physician from having to memorize PJI-optimized thresholds. On the other hand, alpha-defensin may have a higher cost than traditional tests and may not be available in some regions globally. SF-WBC and SF-PMN% are preoperative tests that demonstrate equivalent accuracy to the physicians participating in this study. The main advantage of these tests is that they are inexpensive due to their performance as a multipurpose test at most institutions. Unfortunately, these tests also may exhibit variability across laboratories [10,17] and require the physician to choose the appropriate PJI-optimized threshold to interpret the result, which may result in user error [10]. 

There are limitations to this study. First, although the physicians chosen to participate in this study were specifically chosen for their diversity in geography and specialty, it is possible that the 12 physicians do not represent physicians in general. Given that the physicians chosen to be in this study frequently care for PJI, we do not believe that this study underestimates PJI scoring system usage. Second, different results may have been generated by using a gold standard other than the 2013 MSIS criteria, which we utilized given its widespread use in the literature and the availability of the clinical vignettes used in this study. However, we believe that the use of the 2018 ICM definition or the 2021 EBJIS definition of PJI would have led to even better performance of the individual tests for PJI, relative to physicians, given the prominence of synovial fluid tests, including alpha-defensin in these scoring systems. Third, the results of this study would be irrelevant if all physicians or health systems incorporated algorithmic diagnostic practices, which we believe is very unlikely given the history of non-compliance with CPGs among physicians [5,6].

## Conclusions

Although multiple-criterion scoring systems may outperform individual tests for diagnosing PJI in the research setting, it appears that the complexity of using multiple tests to diagnose PJI causes indecision and variability in diagnosis among physicians. This study, along with previous, existing literature suggests that PJI scoring systems may not function as well in routine clinical use as they do in research, where the thresholds for all tests and the strategy of combining tests can be monitored by a research team.

Physicians’ routine use of multiple preoperative criteria to diagnose PJI is less accurate than the strict algorithmic calculation of the diagnosis as achieved in research. In fact, most physicians in this study would have improved their diagnostic accuracy for PJI by simply utilizing a single good test to make the diagnosis, instead of trying to combine multiple tests into a decision. We propose that less complex diagnostic criteria should be explored for routine clinical utilization.
